# Regulation of Hepatic Stellate Cells and Fibrogenesis by Fibroblast Growth Factors

**DOI:** 10.1155/2016/8323747

**Published:** 2016-09-06

**Authors:** Justin D. Schumacher, Grace L. Guo

**Affiliations:** Department of Pharmacology and Toxicology, Rutgers University, Piscataway, NJ 08854, USA

## Abstract

Fibroblast growth factors (FGFs) are a family of growth factors critically involved in developmental, physiological, and pathological processes, including embryogenesis, angiogenesis, wound healing, and endocrine functions. In the liver, several FGFs are produced basally by hepatocytes and hepatic stellate cells (HSCs). Upon insult to the liver, expression of FGFs in HSCs is greatly upregulated, stimulating hepatocyte regeneration and growth. Various FGF isoforms have also been shown to directly induce HSC proliferation and activation thereby enabling autocrine and paracrine regulation of HSC function. Regulation of HSCs by the endocrine FGFs, namely, FGF15/19 and FGF21, has also recently been identified. With the ability to modulate HSC proliferation and transdifferentiation, targeting FGF signaling pathways constitutes a promising new therapeutic strategy to treat hepatic fibrosis.

## 1. Introduction

Hepatic fibrosis is the result of tissue repair following chronic injury leading to the accumulation of connective tissue within the liver. The primary producers of the connective tissue in a fibrotic liver are hepatic stellate cells (HSCs). During liver injury, HSCs migrate to the location of damage, transdifferentiate into an activated phenotype, produce extracellular matrix to contain the area of injury, and release growth factors to stimulate liver regeneration to replace the damaged tissue. Upon resolution of injury, HSCs undergo apoptosis or revert back to a quiescent phenotype. Chronic liver injury, however, leads to the persistent activation of HSCs, accumulation of extracellular matrix, and eventual development of hepatic fibrosis [1]. HSC activation during liver injury is induced by the paracrine stimulation of HSCs by the surrounding cells/factors in the liver such as hepatocytes, Kupffer cells, endothelial cells, leukocytes, and platelets. The stimuli released by these neighboring cells that regulate HSC activities and proliferation include cytokines, lipid peroxides, growth factors, and reactive oxygen species [1]. This review will focus on an important family of growth factors, fibroblast growth factors (FGFs), which have been shown to regulate HSCs in an autocrine, paracrine, and endocrine fashion.

There are seven subfamilies of FGFs within the FGF family of growth factors. These consist of the FGF1 subfamily (FGF1, FGF2), FGF4 subfamily (FGF4, FGF5, and FGF6), FGF10 subfamily (FGF3, FGF7, FGF10, and FGF22), FGF8 subfamily (FGF8, FGF17, and FGF18), FGF9 subfamily (FGF9, FGF16, and FGF20), FGF11 subfamily (FGF11, FGF12, FGF13, and FGF14), and FGF19 subfamily (FGF15, FGF19, FGF21, and FGF23) [2]. These subfamilies of FGFs have tissue specific expression, varying binding affinity for each fibroblast growth factor receptor (FGFR), and require different cofactors for receptor binding. A large degree of promiscuity has been identified in FGF activation of FGFRs allowing for redundancy in several biological systems [2]. All but one subfamily of FGFs are heparin binding proteins, which limits their functions to autocrine and paracrine signaling [3]. The FGF19 subfamily of FGFs has reduced affinity for heparin allowing their members to circulate systemically and bind FGFRs in distant organs, thereby acting as endocrine factors [4]. Heparin is also the binding cofactor required for activation of FGFRs, except for the FGF19 subfamily [3]. The cofactor required for FGFs of the FGF19 subfamily to activate FGFRs are the klotho proteins. There are two forms of klothos, *α*-klotho and *β*-klotho. The tissue specific expression of these klotho proteins controls the tissue specific effects of the endocrine FGFs [2, 4].

**Figure 1:**
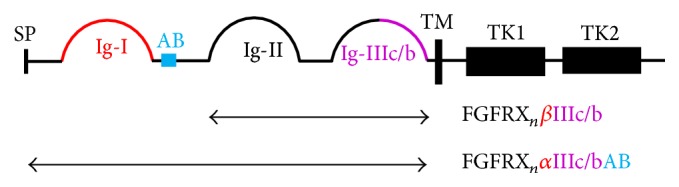
*Structure of fibroblast growth factor receptors*. Ig-I domain is present in *α* but not *β* variants (red). Splice variation in the Ig-III loop distinguishes b and c type receptors (purple). Acid box is present in AB variants (blue). AB: acid box, Ig: immunoglobulin-like domain, SP: signal peptide, TM: transmembrane domain, and TK: tyrosine kinase domain.

There are four isoforms of FGFRs: FGFR1, FGFR2, FGFR3, and FGFR4. The general structure of FGFRs consists of 3 extracellular domains: Ig-like domains (an acid box between the first two Ig-like domains), a transmembrane domain, and two intracellular tyrosine kinase domains (Figure 1) [2]. The first Ig loop in FGFRs is not necessary for ligand binding and actually suppresses FGF and heparin sulfate binding affinity to ligand binding domain located in the second and third Ig loops [5, 6]. Two forms of FGFRs are synthesized: an *α* form possessing the first Ig-like domain and a *β* form that lacks the first Ig-like domain. There are also variant forms of FGFRs that lack the acid box. FGFRs with the acid box present are designated with an AB (e.g., FGFR1*β*IIIcAB). The third Ig-like domain, Ig-III, in FGFR1, FGFR2, and FGFR4 can undergo alternative splicing resulting in two variant Ig-III loops, IIIb and IIIc [7, 8]. The third Ig-like domain in FGFR4 does not undergo alternative splicing [9]. The IIIb and IIIc FGFR splice variants display tissue specific expression. During organogenesis, IIIb FGFRs are expressed by the developing epithelium, whereas IIIc receptors are expressed by the underlying mesenchymal layer. FGF factors produced by the epithelium activate the IIIc isoforms present in mesenchyme while the FGFs produced by the mesenchyme activate the IIIb FGFRs on the epithelium [10–12]. This acts as a paracrine axis controlling organogenesis. This axis is similar to the paracrine axis observed during liver injury in which there is coordinated regulation of FGFR activation on HSCs and hepatocytes by subsequent FGFs; FGFs produced by HSCs activate FGFRs on hepatocytes and hepatocyte-derived FGFs activate FGFRs on HSCs. The autocrine, paracrine, and endocrine effects of FGFs on HSCs and the development of hepatic fibrosis will be reviewed in the sections below and further summarized in Table 1. The intracellular signaling subfamily of FGFs, FGF11 subfamily, will not be discussed in this manuscript as no studies could be identified investigating intracellular FGF signaling in HSC.

## 2. FGFR Expression on HSCs

A systematic survey of FGFR expression was performed in freshly isolated primary rat HSCs [13]. Primers were developed for RT-qPCR that could detect the various splice variants of each FGFR isoform. As may be expected for a mesenchymal cell, HSCs were not found to express FGFR1IIIb, FGFR2IIIb, or FGFR3IIIb. However, HSCs did express the IIIc alternatively spliced isoforms of FGFR1, FGFR2, FGFR3, and FGFR4. Three variants of FGFR1IIIc were expressed: FGFR1*β*IIIcAB, FGFR1*α*IIIc, and FGFR1*α*IIIcAB. The predominant variant was FGFR1*β*IIIcAB. Three variants of FGFR2IIIc were present including FGFR2*β*IIIc, FGFR2*β*IIIcAB, and FGFR2*α*IIIcAB with the primary form expressed being FGFR2*β*IIIc. Only 1 variant of FGFR3, FGFR3*α*IIIcAB, was present. This study only looked at expression of FGFRs in freshly isolated rat HSCs or HSCs cultured only for three days and not in activated HSCs. This is important as FGFR expression may alter upon activation. A separate study determined that FGFR4 expression is upregulated 2.47-fold in Lx-2, a human HSC cell line, upon hypoxia induced transdifferentiation [14]. It is important to note that the above survey of FGFR expression in HSC was only performed in rats, and, to the best of our knowledge, no similar studies have been performed to extensively characterize FGFR variant expression in HSCs of other species. FGFR1 and to a much lesser extent FGFR4 have been shown to be expressed in isolated mouse HSCs [15].

## 3. Autocrine and Paracrine Actions of FGFs on HSCs

Several studies have shown that liver injury and* in vitro* transdifferentiation stimulate HSC production of FGFs including FGF2 [8, 12, 13, 15, 16], FGF7 [17–19], and FGF9 [8]. FGF2 and FGF9 are also expressed basally by hepatocytes. The localized production of FGFs allows for potentially both autocrine and paracrine stimulation of FGFRs at the foci of liver damage. As described below, FGF signaling during liver damage enhances liver regeneration but chronic production can also lead to the development of fibrosis.

### 3.1. FGF1 Subfamily

The members of the FGF1 subfamily, FGF1 and FGF2, have been investigated for their effects on hepatic fibrosis and HSC activation and proliferation. Though all studies have found that FGF1 or FGF2 regulates HSC function or proliferation, there are several conflicting reports. For example, some of the studies described below state that FGF2 does not affect alpha smooth muscle actin (*α*SMA) expression or HSC proliferation whereas other studies state that FGF2 upregulates *α*SMA and induces proliferation. Below are summaries of the key investigations into the effects of FGF1 and FGF2 on HSC function.

Lin et al. determined that primary rat HSCs spontaneously activated over 16 days of culturing produce FGF2 [16]. This study also demonstrates that FGF2 induces the production of collagen 1*α*1 and *α*SMA* in vitro*. FGF2 treatment of HSCs led to increased proliferation indicated by increased incorporation of BrdU. The effects on proliferation were determined to be induced by the activation of the MEK/ERK signaling pathway and altered expression of cyclin D and p21. The effects on HSC proliferation and gene expression by FGF2 were reversible by treatment with NP603, an inhibitor of FGFR1. This study also demonstrates that* in vivo* NP603 was found to ameliorate the upregulation of collagen 1*α*1 and *α*SMA in rats treated with carbon tetrachloride (CCl_4_) [16].

Corresponding to the FGF2 induced proliferation of primary rat HSCs in Lin et al., FGF2 was also shown to act as a mitogen for Lx-2 cells [20]. The induction of Lx-2 proliferation by FGF2 was inhibited by cotreatment with brivanib, an ATP-competitive inhibitor of FGFR, vascular endothelial growth factor receptor, and platelet-derived growth factor receptor [21]. Transforming growth factor beta (TGF*β*) induction of *α*SMA in Lx-2 cells was not inhibited by brivanib indicating that FGF signaling does not affect TGF*β* activation of HSCs. The effects of brivanib on hepatic fibrosis were tested in three animal models, CCl_4_, bile-duct ligation, and thioacetamide fibrosis models, with results showing that brivanib decreased *α*SMA and collagen 1*α*1 expression. Unfortunately, isolating the role of FGF signaling in these animal studies is confounded by the lack of target specificity of brivanib [20].

Juxtaposed to the studies by Lin et al. and Nakamura et al., a study using FGF1 knockout mice (FGF1^−/−^), FGF2 knockout mice (FGF2^−/−^), and FGF1 and FGF2 double knockout mice (FGF1^−/−^FGF2^−/−^) found that FGF1 and FGF2 regulated the expression of collagen 1*α*1 but does not affect HSC migration or proliferation [22]. In this study, groups of 8-week-old male FGF1^−/−^, FGF2^−/−^, and FGF1^−/−^FGF2^−/−^ mice were treated with CCl_4_ acutely with one dose or chronically for 3 weeks. In both the acute and chronic study, it was found that the FGF1^−/−^, FGF2^−/−^, and FGF1^−/−^FGF2^−/−^ mice had attenuated expression of collagen 1*α*1 but no effects on *α*SMA expression were seen. The extent and time course of TGF*β* expression upon injury were not altered in the FGF1^−/−^FGF2^−/−^, indicating that the mechanism by which these FGFs regulate the development of fibrosis is not through mitigation of TGF*β* expression. Desmin expression, a surrogate estimator for the number of HSCs present in the liver, was similar between wild type and FGF1^−/−^FGF2^−/−^ mice. Therefore, the authors concluded that FGF1 and FGF2 do not regulate HSC proliferation. This finding is in congruence with an* in vitro* study using both primary rat HSCs and Lx-2 cells [13]. During this study, HSCs were treated with FGF2 and cell proliferation was measured by ^3^H-thymidine DNA incorporation. Though FGF2 was found to lead to the phosphorylation of ERK1, ERK2, JNK1, and JNK2/3, FGF2 did not alter HSC proliferation.

The studies described above all briefly investigated the interaction between FGF2 and TGF*β* signaling. This interaction was also studied* in vitro* using cultured human myofibroblastic liver cells (MFLCs) [23]. TGF*β* was found to increase the expression of FGF2 and FGFR1 by MFLCs. Treatment of MFLCs with anti-FGF2 antibodies inhibited the proliferative effects of TGF*β* but not the expression of fibronectin. This study concluded that FGF2 acts as an autocrine factor mediating the proliferative response, but not the profibrotic response, of MFLCs to TGF*β*. This aligns with the finding that FGFR inhibition by brivanib did not modulate *α*SMA expression induced by TGF*β* [20].

In summary, FGF2 derived from HSCs and hepatocytes functions as an autocrine and paracrine signaling molecule regulating HSC function during liver injury. Correspondingly, autocrine stimulation of fibroblasts by FGF2 has also already been implicated as a key mediator of the development of bone marrow [24] and lung fibrosis [17]. In addition to its autocrine effects, HSC-derived FGF2 also functions in a paracrine manner to induce hepatocyte growth and regeneration during injury. It has been well studied that FGF2 is a strong proliferative signal for hepatocytes [18, 19, 25]. After partial hepatectomy, injection with FGF2 increased uptake of ^3^H-thymidine in the liver [19]. FGF2 is also required for the proper organization of cells within the liver, as FGF2 deficient mice that underwent partial hepatectomy had altered liver structures after regeneration [25].

### 3.2. FGF7 Subfamily

The production of FGF7 by HSCs during liver injury has also been investigated. In two human studies, livers were collected from patients with cirrhosis, hepatitis B and hepatitis C (HBV, HCV), autoimmune hepatitis, and alcohol induced liver damage [26, 27]. Both studies found that FGF7 was expressed in fibrotic livers but not in healthy control liver samples. Steiling et al. noted that the fibrosis staging in HCV patients was positively correlated with FGF7 mRNA levels and immunohistochemistry (IHC) of the liver showed that FGF7 expression colocalized with *α*SMA [26]. Otte et al. also included an animal study parallel to their clinical investigation [27]. In detail, the male Wistar rats were exposed to phenobarbitone and CCl_4_ for up to 70 days. In congruence with the human clinical data, IHC of liver sections from the treated rats revealed that FGF7 was exclusively expressed in myofibroblasts in fibrotic foci with isolated protein and mRNA levels of FGF7 positively correlated to fibrosis severity [27]. The function of HSC-derived FGF7 has been explored in a mouse partial hepatectomy model [28]. HSCs from hepatectomized mice had a 3.3-fold increase in FGF7 expression compared to HSCs from sham mice. Expression of FGFR2b, the receptor for FGF7, was found to increase 3-fold after partial hepatectomy, with IHC revealing strongest staining in hepatocytes. To perform a gain-of-function study, a group of mice was given a hydrodynamic tail vein injection of plasmid encoding a HA-tagged FGF7. Overexpression of FGF7 led to an accelerated incorporation of BrdU and expression of PCNA in hepatocytes after partial hepatectomy. These data indicate that the upregulation of FGF7 in HSCs and upregulation of FGFR2b on hepatocytes act as a paracrine axis driving hepatocyte regeneration after liver injury. No studies could be identified in which the direct effect of FGF7 on HSC activation or proliferation was investigated. However, it has been shown that HSCs in rats do not express FGFR2b but, instead, express FGFR2c that is not activated by FGF7 [13]. Thus it is unlikely that FGF7 will greatly affect HSCs directly.

### 3.3. FGF9 Subfamily

FGF9 has also been found to be expressed by HSCs [13]. Liver slices were cultured with or without CCl_4_ treatment. IHC of the untreated cultured liver slices indicates that FGF9 is basally expressed in hepatocytes and a few HSCs. Upon treatment with CCl_4_ the number of FGF9-positive HSCs was greatly increased. FGF9 expression was measured in isolated primary rat HSCs before and after activation. Upon transdifferentiation into an activated phenotype, HSCs upregulate FGF9 expression 5- to 10-fold. Expression of FGF16 and FGF20, the two other members of the FGF9 subfamily, was not detected in HSCs by RT-qPCR. The authors noted that, though HSCs express the receptors activated by FGF9, FGF9 failed to induce HSC proliferation measured by ^3^H-thymidine incorporation. However, FGF9 did act as a mitogen for hepatocytes [13]. Hence, similar to FGF2 and FGF7, FGF9 produced by HSCs functions to increase hepatocyte proliferation and regeneration upon injury.

## 4. Regulation of HSCs and Fibrosis by Endocrine FGFs

The endocrine subfamily of FGFs consists of FGF19, FGF15 (the mouse homolog of human FGF19), FGF21, and FGF23. There are now several studies that have investigated the endocrine functions of FGF15/19, FGF21, and FGF23. FGF15/19 is produced in the ileum and partially in the liver (FGF19 only) and has been shown to act as a negative feedback factor suppressing bile acid synthesis by hepatocytes [29–32]. FGF15/19 signaling has also been found to affect insulin sensitivity, serum lipid levels, weight loss, energy homeostasis, and cell proliferation [4, 33–35]. FGF21 is highly expressed in the liver and functions to regulate glucose and lipid metabolism [36]. FGF23 is involved in a bone-kidney axis and regulates bone mineralization, vitamin D homeostasis, and systemic phosphate levels [37].

The effects of FGF15/19 and FGF21 on hepatic fibrosis are now emerging. Several clinical studies have now been performed identifying the correlation of serum and liver concentrations of endocrine FGFs to various forms of hepatic fibrosis. A few animal studies have also now been published identifying the mechanisms by which FGF15/19 and FGF21 mediate the development of hepatic fibrosis. The findings from these studies are described in the following sections.

### 4.1. FGF15/19

The effect of FGF15 on CCl_4_ induced liver fibrosis has recently been investigated [15]. Mice were injected intraperitoneally (IP) with the carcinogen diethylnitrosamine at the age of 15 days and were subsequently given biweekly IP injections of CCl_4_. After 27 weeks, FGF15 deficient mice were found to have decreased hepatic fibrosis compared to wild type (WT). In congruence with histologic findings, FGF15 knockout mice were found to have downregulated collagen 1*α*1, tissue inhibitor of metalloproteases 1, *α*-SMA, and connective tissue growth factor (CTGF) compared to wild type. The induction of TGF*β* observed in wild type mice treated with CCl_4_ was not observed in knockout mice. Overexpression of FGF15 using an adenovirus vector led to roughly 3-fold elevations of both hepatic TGF*β* and CTGF expression. Using* in vitro* experiments, this study proposed that FGF15 affected HSCs indirectly; specifically, FGF15 signaling increases CTGF release from hepatocytes leading to the paracrine activation of HSCs. Treatment of isolated mouse HSCs with FGF19 showed no changes in cyclin D or *α*SMA expression [15]. Though no effects were seen during this study, a direct effect of FGF15/19 on HSCs should not be ruled out. As FGFR4 is upregulated over 2-fold in HSCs upon activation [14], FGF15/19 signaling may be enhanced in activated HSCs. Additionally, this study treated mouse HSCs with human FGF19 and therefore FGF19 may have failed to activate the mouse receptor efficiently.

Recently, many clinical studies have found correlations between serum FGF19 levels and severity of hepatic fibrosis of multiple etiologies. However, whether FGF19 serum levels were positively or negatively correlated to fibrosis score depended upon the etiology of disease. This may be attributed to the fact that FGF19 may affect disease pathogenesis via regulation of bile acid levels or through regulation of activation of HSCs. For this reason, understanding of disease progression is extremely important when considering the reasons underlying FGF19 and fibrosis correlations.

Severity of lobular and portal fibrosis in patients with pediatric onset intestinal failure was found to be negatively correlated to FGF19 levels [38]. Serum concentrations of the inflammatory markers and portal inflammation severity were also negatively correlated to FGF19 serum concentrations. Of the 42 patients screened, 57% were found to have serum bile levels out of range. As FGF19 is a negative feedback factor for bile acid synthesis, the observed hepatic fibrosis and inflammation in patients with low serum concentrations of FGF19 may have been the result of dysregulated bile acid production and bile acid toxicity. The pattern of portal fibrosis and inflammation observed in these patients is in agreement with this hypothesis. Similar results have been found in an experiment model of short bowel syndrome. Bowel resection in piglets led to an altered microbiome, altered bile acid pool composition, altered farnesoid X receptor activation, and failure of hepatic small heterodimer protein to downregulate bile acid synthesis [39]. The authors proposed that the accumulation of hepatic bile acids led to the observed liver damage.

A recent study also examined the use of serum and liver FGF19 levels as a biomarker for severity of primary biliary cirrhosis (PBC) [40]. This study found that serum FGF19 levels were positively correlated to Mayo Risk Score for PBC. This paper demonstrates that FGF19 is expressed 9-fold greater in the liver of noncirrhotic PBC patients compared to healthy individuals and 69-fold greater in the liver of cirrhotic PBC patients. In patients with fibrosis, higher hepatic FGF19 mRNA levels were associated with worsened fibrosis severity. Hepatocytes with upregulated FGF19 were also found to induce FGFR4 expression. Therefore, the authors proposed that the production of FGF19 during PBC is a compensatory mechanism to decrease bile production in an autocrine fashion [40].

### 4.2. FGF21

The role of FGF21 in the regulation of HSC activation, apoptosis, and development of fibrosis has been reported in both gain-of-function and loss-of-function studies. In the gain-of-function study, male ICR mice were given 10 mg/kg dimethylnitrosamine (DMN) for the first three consecutive days of each week for 4 weeks [41]. FGF21 was given to the mice every 12 hours after DMN treatment. Animals receiving exogenous FGF21 treatment had reduced fibrosis and attenuated induction of collagen 1*α*1, *α*SMA, and TGF*β* protein and mRNA levels. TGF*β* signaling was also altered with FGF21-treated mice having decreased pSmad2/3 : Smad2/3 ratio. FGF21 seems to be protective against the development of hepatic inflammation as protein and mRNA levels of inflammatory molecules, TNF*α*, IL-6, and IL-1*β*, were reduced as well as the pI*κ*B/I*κ*B and p65/lamin b1 ratios.* In vitro* treatment of T6 cells, a rat HSC cell line, with FGF21 was performed in the presence of ethanol or platelet-derived growth factor (PDGF). FGF21 decreased collagen 1*α*1, *α*SMA, and TGF*β* expression induced by both ethanol and PDGF. FGF21 was also found to be proapoptotic by reducing Bcl-2 : Bax ratios.

The effect of FGF21 on fibrosis was also studied in a loss-of-function study model. Wild type and FGF21 deficient mice were fed a methionine and choline deficient diet (MCDD) for 8 to 16 weeks [42]. The FGF21 deficient mice were found to have worsened steatosis, inflammation, and fibrosis. Collagen 1*α*1, *α*SMA, and TGF*β* mRNA levels were elevated in the knockout mice in addition to the expression of genes involved in inflammation and fatty acid transport: monocyte chemoattractant protein 1, macrophage inflammatory protein 1 alpha, IL-1*β*, and CD36. The altered expression of all of the previously mentioned genes was reversible by continuous subcutaneous infusion of FGF21 to the FGF21 deficient mice.

Despite the protective nature of FGF21 in animal models, many clinical studies have reported a positive correlation between steatosis and fibrosis severity and serum FGF21 levels in humans [43–47]. Due to the correlations found in these studies, it has been proposed that serum FGF21 levels can be used as a biomarker for nonalcoholic fatty liver disease, nonalcoholic steatohepatitis, and other liver pathologies. As FGF21 is predominantly produced in the liver it is probable that the increased FGF21 serum levels observed in these studies is due to a compensatory increase in hepatic FGF21 production to attempt to mitigate liver injury.

## 5. Conclusions

FGFs play an important role in the development of hepatic fibrosis acting as autocrine, paracrine, and endocrine mediators of hepatocyte regeneration and HSC migration, proliferation, and transdifferentiation. The various subfamilies of FGFs have been shown to affect fibrogenesis by different mechanisms. The studies described in this review were either performed in whole body knockout mice or using pharmacologic manipulation with compounds of varying specificity for each FGFR. To the authors' knowledge, no studies have been published using HSC-specific FGF or FGFR knockout mice. Conditional knockout mice with floxed FGF and FGFR genes are available and therefore creation of HSC-specific knockout of FGF and FGFR in mice is possible [48–51]. Studies using these mice could provide further insight into FGF regulation of HSCs.

With the growing understanding of the mechanisms by which FGFs regulate hepatic fibrosis, many clinical applications targeting the FGF pathway are emerging. The ability of FGFs to regulate HSC proliferation, migration, and transdifferentiation makes FGF signaling an attractive pathway to target for the treatment of hepatic fibrosis. Therapeutic agents are now in development which target many levels of this pathway: inhibition of FGFRs [52], sequestration of FGFs [53], modulation of FGF expression [54], and treatment with recombinant FGF protein [55]. In addition to acting as a therapeutic target, evidence for the use of serum FGF concentrations as clinical biomarkers for many liver diseases is growing [38, 40, 43–47]. When using serum FGF levels as a biomarker it is important to consider the disease pathogenesis and to understand the reason underlying the correlation of serum FGF levels to disease severity; are the serum FGF concentrations influencing disease severity or is disease severity influencing the production of FGF? Whether used as a clinical biomarker or targets of therapeutic agents, FGFs may have an expanding role in the management of hepatic fibrosis.

## Figures and Tables

**Table 1:** Regulation of HSCs and development of hepatic fibrosis by various FGF isoforms.

FGF subfamily	Study	Findings
FGF1 subfamily	Lin et al. [16]	FGF2 induces collagen 1*α*1 and *α*SMA in HSCs *in vitro*. FGF2 increases HSC proliferation mediated by MEK/ERK signaling.

FGF1 subfamily	Nakamura et al. [20]	FGF2 increases proliferation of Lx-2 cells. Inhibition of FGFR1 does not alter *α*SMA induction in Lx-2 cells treated with TGF*β*.

FGF1 subfamily	Yu et al. [22]	Single and double knockout of FGF1 and FGF2 decreases CCl_4_ induced hepatic fibrosis. Single and double FGF1 and FGF2 knockout mice have reduced collagen 1*α*1 expression but not *α*SMA. Desmin expression in the liver remained constant in FGF1 and FGF2 deficient mice indicating FGF1 and FGF2 do not affect HSC proliferation.

FGF1 subfamily	Antoine et al. [13]	FGF2 treatment fails to affect the proliferation of Lx-2 cells or primary rat HSCs *in vitro*.

FGF1 subfamily	Rosenbaum et al. [23]	TGF*β* increases the expression of FGF2 by MFLCs. FGF2 mediates TGF*β* induced HSC proliferation but does not alter expression of fibronectin.

FGF7 subfamily	Steiling et al. [26]	FGF7 is expressed in fibrotic livers but not healthy controls. FGF7 expression is colocalized with *α*SMA in stained liver sections.

FGF7 subfamily	Otte et al. [27]	IHC of liver sections from rats treated with phenobarbitone and CCl_4_ revealed FGF7 is exclusively expressed by HSCs in fibrotic foci. Severity of hepatic fibrosis correlated positively to FGF7 expression.

FGF7 subfamily	Tsai and Wang [28]	FGF7 accelerates DNA incorporation of BrdU and expression of PCNA in hepatocytes after partial hepatectomy.

FGF9 subfamily	Antoine et al. [13]	FGF9 is expressed in hepatocytes and HSCs basally but is greatly upregulated in HSCs after CCl_4_ exposure. FGF16 and FGF20 expression has not been detected in HSCs. FGF9 induces hepatocyte proliferation but not HSC proliferation.

FGF19 subfamily	Uriarte et al. [15]	FGF15 deficiency reduces hepatic fibrosis in mice treated with DEN and CCl_4_. Collagen 1*α*1, Timp1, *α*SMA, and CTGF expression induced by CCl_4_ is mitigated in FGF15 deficient mice. CCl_4_ treatment of transgenic mice overexpressing FGF15 have 3-fold higher expression of TGF*β* and CTGF compared to WT mice. FGF15 signaling increases CTGF release from hepatocytes leading to the paracrine activation of HSCs.

FGF19 subfamily	Xu et al. [41]	DMN treated mice cotreated with FGF21 have had reduced fibrosis and mitigated expression of collagen 1*α*1, *α*SMA, and TGF*β*. FGF21 reduced TGF*β* signaling is observed as a decrease in pSmad2/3 : Smad2/3 ratio. FGF21 attenuates DMN induced hepatic inflammation and reduces TNF*α*, IL-6, and IL-1*β* expression. *In vitro* treatment of T6 cells with FGF21 decreases alcohol and PDGF inductions of collagen 1*α*1, *α*SMA, and TGF*β*. FGF21 reduces Bcl-2/Bax ratio *in vitro* in cultured T6 cells.

FGF19 subfamily	Fisher et al. [42]	FGF21 deficient mice fed a MCDD have increased fibrosis with increased expression of collagen 1*α*1, *α*SMA, and TGF*β*. MCDD fed FGF21 knockout mice have increased hepatic inflammation with increased expression of MCP-1, MIP1*α*, IL-1*β*, and CD36. The increased expression of profibrotic and proinflammatory genes in FGF21 deficient mice is reversible by continuous subcutaneous infusion of FGF21.

## Abbreviations

*α*SMA: Alpha smooth muscle actin
CCl_4_: Carbon tetrachloride
CTGF: Connective tissue growth factor
DMN: Dimethylnitrosamine
FGF: Fibroblast growth factor
FGFR: Fibroblast growth factor receptor
HSCs: Hepatic stellate cells
IHC: Immunohistochemistry
IP: Intraperitoneal
MFLCs: Myofibroblastic liver cell
PBC: Primary biliary cirrhosis
PDGF: Platelet-derived growth factor
TGF*β*: Tumor growth factor beta.
